# Wheat Flour, Enriched with γ-Oryzanol, Phytosterol, and Ferulic Acid, Alleviates Lipid and Glucose Metabolism in High-Fat-Fructose-Fed Rats

**DOI:** 10.3390/nu11071697

**Published:** 2019-07-23

**Authors:** Xiao-Xuan Guo, Zhu Zeng, Yong-Zhong Qian, Jing Qiu, Kai Wang, Yong Wang, Bao-Ping Ji, Feng Zhou

**Affiliations:** 1 Institute of Quality Standard and Testing Technology for Agro-products, Chinese Academy of Agricultural Sciences, Beijing 100081, China; 2 Beijing Key Laboratory of Functional Food from Plant Resources, College of Food Science and Nutritional Engineering, China Agricultural University, Beijing 100083, China; 3 State Key Laboratory of Silkworm Genome Biology, Key Laboratory of Sericulture Biology and Genetic Breeding, Ministry of Agriculture and Rural Affairs, College of Biotechnology, Southwest University, Chongqing 400715, China; 4 Institute of Apicultural Research, Chinese Academy of Agricultural Sciences, Beijing 100093, China; 5 Academy of State Administration of Grain, Beijing 100037, China

**Keywords:** lipid/glucose metabolism, γ-oryzanol, phytosterol, ferulic acid, wheat flour

## Abstract

(1) Background: Modern dietary patterns with a high intake of fat and fructose, as well as refined carbohydrates, closely relate to lipid/glucose metabolic disorders. The main objective of this study is to provide new thoughts in designing functional food with some lipid/glucose metabolism regulating effects for obese people. (2) Methods: The alleviating abilities of γ-oryzanol, phytosterol or ferulic acid-enriched wheat flour on lipid/glucose metabolic dysfunction were evaluated in male SD rats induced by a high-fat-fructose diet. The underlying mechanisms were clarified using western blot. (3) Results: In an in vitro cell model, γ-oryzanol, phytosterol and ferulic acid regulate lipid/glucose metabolism by increasing the phosphorylation of AMPK and Akt, and PI3K expression, as well as decreasing expressions of DGAT1 and SCD. The in vivo study shows that ferulic acid and γ-oryzanol-enriched flours are beneficial for managing body weight, improving glucose metabolism, hyperlipidemia and hepatic lipid accumulation. Phytosterol-enriched flour exerted remarkable effects in regulating hyperinsulinemia, insulin resistance and hyperuricemia. Western blot analysis of proteins from liver samples reveals that these enriched flours alleviated hepatic lipid accumulation and insulin resistance through their elevation in the phosphorylation of AMPK and Akt. (4) Conclusions: Our study indicates that these enriched flours can serve as a health-promoting functional food to regulate obesity-related lipid/glucose metabolic dysfunction in rats.

## 1. Introduction

Obesity is one of the leading worldwide metabolic disorders that is characterized by excessive accumulation of the adipose tissue [1]. The prevalence of obesity is associated with the onset of several pathological conditions, such as type 2 diabetes, cardiovascular disease and dyslipidemia [2]. Several therapeutic strategies have been developed in order to treat obesity and to reduce the risk factors for metabolic syndrome. These include the inhibition of pancreatic lipase [3], improvement in glucose and lipid metabolic homeostasis [4], etc.

Consumption of refined wheat flour is widespread throughout the world. However, the World Health Organisation (WHO) recommends restricting the intake of refined carbohydrates, since their consumption increases the risk of numerous metabolic disorders [5]. In recent years, whole wheat flour has drawn worldwide attention due to its ability to lower some risk factors for metabolic diseases [6]. However, the rough taste resulting from the high bran content presents a major concern for the consumption of whole wheat flour. Thus, in the present study we utilized 90% wheat flour (90 flour), which is produced by removing 10% of the outer layer and the wheat germ of whole wheat grain, as the base flour to develop the enriched wheat flour.

In recent years, phytochemicals derived from natural food are being increasingly used for preventing obesity and its associated lipid/glucose metabolic disorder due to their natural sources and low side effects. Certain bioactive phytochemicals obtained from the by-products (wheat bran, wheat germ, rice bran, etc.) of the refining process of grains are beneficial for obesity-related metabolic disorders. Ferulic acid is the most abundant phenolic acid in wheat grains that is covalently bonded to arabinoxylans and other polysaccharides or proteins within the cell walls [7,8]. Its antioxidant nature has demonstrated significant improvement in lipid and glucose homeostasis in the obese mouse model and hyperlipidemic subjects [7,9]. Phytosterols are structurally similar to cholesterol that are rich in the by-products of cereals [10]. The US National Cholesterol Education Program (NCEP) recommends that the daily intake of phytosterols as a nutrient supplementation in a dose of 2 g could lower LDL cholesterol and reduce the risks of cardiovascular disease [11]. Another phenolic compound, γ-oryzanol is mainly composed of the esters of trans-ferulic acid with phytosterols. It is found in abundance in rice bran oil that exhibits antioxidant and anti-inflammatory properties, resulting in an improved lipid profile and glucose metabolism in high fat-fed mice [12,13]. Therefore, the present study incorporated ferulic acid, γ-oryzanol, and phytosterol to enrich wheat flour, in an effort to provide new thoughts in designing functional food to regulate metabolic syndrome.

The main cause of obesity is considered to be an imbalance in calorie intake. Westernized diets characterized by high fats, along with an excessive intake of fructose, in beverages and desserts, are probable causes of obesity [14]. It has been demonstrated that a long term intake of fructose can lead to the development of dyslipidemia, insulin resistance and abdominal fat accumulation in humans, as well as experimental animals [15,16,17]. Therefore, the present study used a rat model fed with a high-fat-fructose diet (HFFD) to investigate the effects of enriched wheat flour on glucose and lipid metabolism. Although ferulic acid and phytosterol are the major metabolites of γ-oryzanol [18], few researchers focus on the comparison of them in ameliorating the metabolic syndrome. Thus, in the present study, the effect of γ-oryzanol, ferulic acid and phytosterol in regulating glucose and lipid metabolism and the potential protein targets, were first investigated on a cell model with glucose and lipid metabolic disorder. Then the alleviation effects of the enriched wheat flour were evaluated in rats fed with a western type of HFFD. We anticipate that this study would provide alternative ways to treat and prevent glucose/lipid metabolic disorder and its associated diseases.

## 2. Materials and Methods

### 2.1. Chemicals

γ-Oryzanol, phytosterol, ferulic acid (98% pure) were obtained from Nanjing Jingzhu Bio-technology Co. Ltd. (Nanjing, China). 90 Flour were provided by Shandong Dong’ao Flour Co. Ltd. (Heze, China). Oleic acid, palmitic acid, and insulin were obtained from Sigma-Aldrich, Inc. (St. Louis, USA). Dulbecco’s Modified Eagle Media (DMEM) with/without glucose, phenol red, fetal bovine serum (FBS), penicillin and streptomycin were purchased from Gibco Life Technologies (Grand Island, NY, USA). Free fatty acid (FFA)-free bovine serum albumin (BSA) was obtained from Wako Pure Chemical Industries, Ltd. (Osaka, Japan). Nitrocellulose membrane was purchased from Bio-Rad Laboratories, Inc. (Hercules, CA, USA). Polyvinylidene difluoride (PVDF) membrane was purchased from Merck KGaA (Darmstadt, Germany). BSA for blocking was purchased from VWR International, LLC. (Radnor, PA, USA). 

Primary antibodies against β-actin, tubulin, AMP-activated protein kinase α (AMPKα), phospho-AMPKα (Thr172), insulin receptor β (IRβ), PI3 Kinase p110α, Akt (pan), phospho-Akt (Ser473), and glucose transporter type 4 (GLUT4) were purchased from Cell Signaling Technology (Danvers, MA, USA). Diacylglycerol acyltransferase 1 (DGAT1) and Stearoyl-CoA desaturase (SCD) were bought from Santa Cruz Biotechnology (Santa Cruz, CA, USA). Horseradish peroxidase (HRP)-linked anti-mouse antibody and HRP-linked anti-rabbit antibody were purchased from Cell Signaling Technology (Danvers, MA, USA). Pierce ECL Western Blotting Substrate was purchased from Thermo Fisher Scientific Inc. (Waltham, MA, USA). BCIP/NBT alkaline phosphatase color development kit was bought from Beyotime Biotechnology (Shanghai, China). All other chemicals used were of analytical grade.

### 2.2. Cell Culture and Treatments

HepG2 cells were purchased from the American Type Culture Collection (Manassas, VA, USA), cultured in DMEM supplemented with 10% FBS and 1% penicillin-streptomycin solution. The lipid/glucose metabolic dysfunction cell model was induced using an induction media containing 1 mM of FFA (oleic and palmitic acid (2:1) in incomplete glucose-free DMEM containing 1% BSA), 10 mM glucose and 15 mM fructose [19]. After attachment into a 96 well plate for 24 h, HepG2 cells were treated with induction media with or without functional components for 24 h. Concentration for each individual compound (γ-oryzanol, phytosterol, ferulic acid) were 100 μM, which were shown to be non-toxic by MTT assay. For the determination of cellular triglyceride (TG) and malonaldehyde (MDA) levels, the cells were rinsed with PBS and lysed with RIPA buffer after treatments. The cell lysates were used for the determination of TG and MDA levels using commercial kits (Nanjing Jiancheng Bioengineering Inst., Nanjing, China). For the determination of insulin-induced glucose absorption, the cells were rinsed with PBS after treatment and treated with incomplete DMEM (high glucose, no phenol red), containing 10-9 M insulin. After 24 h, the glucose level in supernatants were determined using a commercial kit (Nanjing Jiancheng Bioengineering Inst., Nanjing, China). The absorption rates were calculated by comparing with the level in incomplete DMEM (high glucose, no phenol red). The results of cellular TG, MDA levels and glucose absorption in model and treated cells, were expressed by comparing with the levels in control cells.

### 2.3. Western Blot Analysis of Protein from Cells

After treatment, proteins from HepG2 cells were extracted and quantified using commercial kits (Dalian Meilun Biotechnology Co. LTD., Dalian, China). Protein (40 μg) was separated on 10% Sodium dodecylsulphate polyacrylamide gel electrophoresis (SDS-PAGE) at a constant voltage of 100 V, and transferred to nitrocellulose membranes under a current of 250 mA for 1.5 h. After blocking with 5% BSA, the membranes were incubated with primary antibodies (β-actin, AMPK, p-AMPK, DGAT 1, SCD, IRβ, PI3K, Akt, p-Akt, and GLUT4) overnight at 4 °C, followed by incubation with the HRP-linked secondary antibody at room temperature for 1 h. The blots were incubated with ECL substrate for 1 min and the signal intensities were visualized by film exposure. Quantification of bands was performed using Image J Software.

### 2.4. Animals and Diet

Sixty male Sprague-Dawley rats (180–200 g) were obtained from Vital River Laboratory Animal Technology Co. Ltd. (Beijing, China). They were individually housed in a room maintained at 23 ± 1 °C with a relative humidity of 50 ± 20% and light/dark cycle of 12 h. At the end of the acclimatization period (1 week), the rats were randomly divided into 6 groups (*n* = 10/group). They were placed on the following diets for 16 weeks: (i) Regular rodent diet (Control, 13.9 kJ/g, 4.5% fat, w/w); (ii) HFFD with refined wheat flour (Model, 18.9 kJ/g, 42% refined wheat flour, 18% fructose and 20% lard); (iii) HFFD with 90 flour (90F, 18.7 kJ/g); (iv) HFFD with γ-oryzanol enriched 90 flour (OZ-90F, 18.6 kJ/g); (v) HFFD with phytosterol enriched 90 flour (PT-90F, 18.6 kJ/g); (vi) HFFD with ferulic acid enriched 90 flour (FA-90F, 18.6 kJ/g).

The composition of diets is presented in Table 1. The γ-oryzanol, phytosterol and ferulic acid were added at the same molar concentration in the 90 flour, with proportions of 0.71%, 0.48% and 0.24%, respectively.

The rats were fed for 16 weeks with diets and water ad libidum. Body weight was measured weekly, and food consumption was monitored daily. At the end of the experimental period, the rats underwent overnight fasting and were sacrificed under deep anesthesia. Liver, epididymal and the perirenal fat pad were dissected and weighed immediately. The protocols were approved by the Ethics Committee of the Beijing Key Laboratory of Functional Food from Plant Resources (Permit number: A330-5) and were conducted in accordance with the guidelines for animal care of the National Institute of Health [20].

### 2.5. Measurement of Serum Biochemical Parameters

Blood samples were collected from the retro-orbital venous sinus at week 4, 8, 12 and 16 from 12-h fasted rats. Serum was obtained by centrifugation of the blood samples at 4000× *g* for 10 min at 4 °C. The concentrations of serum TG, total cholesterol (TC), low density lipoprotein-cholesterol (LDL-C), high density lipoprotein-cholesterol (HDL-C), uric acid (UA), activities of alanine aminotransferase (ALT), aspartate aminotransferase (AST), and alkaline phosphatase (ALP) were measured using commercial assay kits (Biosino Biotechnology and Science Inc., Beijing, China) on an Alcyon 300 automatic analyzer (Alcyon, USA). The MDA levels of the serum were determined with kits purchased from Nanjing Jiancheng Bioengineering Inst. (Nanjing, China). Concentration of fasting blood insulin (FBI) was determined by ELISA kits from the Beijing Sino-uk Institute of Biological Technology (Beijing, China). Fasting blood glucose (FBG) levels were collected from the tail and measured with a glucometer (Life Scan Inc., Milpitas, CA, USA). The homeostasis model of assessment for the insulin resistance index (HOMA-IR) is used to estimate this insulin resistance. It is calculated as follows, HOMA-IR= FBG (mmol/L) × FBI (mU/L)/22.5.

### 2.6. Oral Glucose (OGTT) and Insulin (ITT) Tolerance Tests

OGTT was measured at week 3, 7, 13 and 15. Gavage with glucose (2 g/kg body weight) was conducted on 12-h overnight fasted rats. Tail blood was collected at 0 (before glucose administration), 30, 60, 90, and 120 min using a glucometer. ITT was measured at weeks 5, 9 and 13.

For ITT, the basal blood glucose levels were measured after 4 h of food deprivation before an intraperitoneal injection of insulin (0.5 U/kg body weight). Blood tests were carried out as for the OGTT. The total AUC of OGTT and ITT were calculated using a trapezoidal method.

### 2.7. Measurement of Hepatic Lipid Accumulation

The livers were homogenized followed by the extraction of lipids [21]. Hepatic TG and TC levels were determined in the same way as the serum lipid level. The hepatic MDA levels were determined according to the instructions of kits (Nanjing Jiancheng Bioengineering Inst., Nanjing, China).

### 2.8. Western Blot Analysis of Protein From Liver Tissue

Extraction of whole tissue protein fractions was performed with liver tissue samples as described by Das et al. [22] Concentration of protein was measured by the Bradford method. Equal amount of protein (40 μg) was separated on SDS-PAGE as previously described in 2.3, and transferred to PVDF membranes. After blocking with 5% BSA, the membranes were incubated with primary antibodies (Tubulin, AMPK, p-AMPK, Akt, p-Akt) overnight at 4 °C, followed by incubation with the HRP-linked secondary antibody at room temperature for 1 h. The blots were stained using a BCIP/NBT alkaline phosphatase color development kit. Quantification of bands was performed using Image J Software.

### 2.9. Histological Analysis

Sections of hepatic tissue, pancreas tissue and epididymal fat tissue was fixed in 10% (*v*/*v*) formalin solution and embedded in paraffin wax. Then the tissues were sliced (3 μm) and subjected to hematoxylin and eosin (HE) staining. All of the sections were observed under a BA-9000L microscope (Osaka, Japan)

### 2.10. Statistical Analysis

Results have been presented as means ± SEM. Data differences from animal experiments were evaluated with a one-way analysis of variance (ANOVA), followed by Duncan’s multiple-comparisons test performed by SAS v8.2. Data differences from cell experiments were evaluated with one-way Analysis of Variance (ANOVA), followed by Tukey’s test using Origin 8.0. Differences were considered significant at *p* < 0.05.

## 3. Results

### 3.1. Effect of Functional Compounds on TG, MDA Levels, and Glucose Absorption in HepG2 Cells

**Figure 1:**
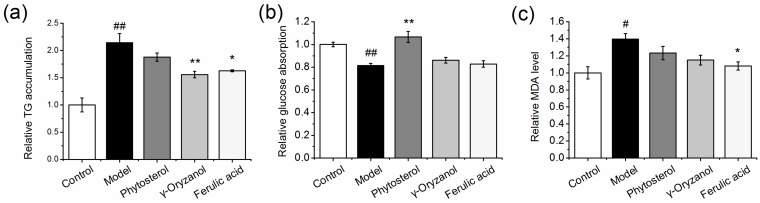
Relative TG accumulation (**a**), glucose absorption (**b**) and malondialdehyde level (**c**) in HepG2 cells. HepG2 cells were treated with an induction medium (10 mM glucose, 15 mM fructose and 1 mM FFA) for 24 h with or without functional components (100 μM). 1% BSA in incomplete DMEM was selected as control. Data are presented as mean ± SEM (*n* = 6). # *p* < 0.05 vs. control. ## *p* < 0.01 vs. control. * *p* < 0.05 vs. model. ** *p* < 0.01 vs. model.

As shown in Figure 1, induction media induced over a 2-fold increase in TG accumulation in model cells compared to control cells. The intracellular TG accumulation was significantly diminished by approximately 25% by γ-oryzanol and ferulic acid treatments, as compared to the model cells (*p* < 0.05), while phytosterol showed a tendency to decrease TG accumulation (*p* > 0.05). The model cells displayed a significantly decreased insulin-induced glucose absorption (*p* < 0.05), indicating an impairment in the insulin signal transduction. Phytosterol exerted the insulin sensitizing effect, since the insulin-induced glucose uptake was significantly improved by 30.8%, compared to model cells (*p* < 0.01), which was even higher than the level of normal cells. As an indicator of lipid peroxidation, the MDA level was detected. Ferulic acid showed the best antioxidant activity among the three compounds, which exhibited a 22.9% decrease in MDA level compared to model cells (*p* < 0.01). γ-Oryzanol and phytosterol also showed a tendency in the decrease of the MDA level, however the effect was not statistically significant (*p* > 0.05).

### 3.2. Expression of Lipid/Glucose Metabolism-Related Proteins in HepG2 Cells

**Figure 2:**
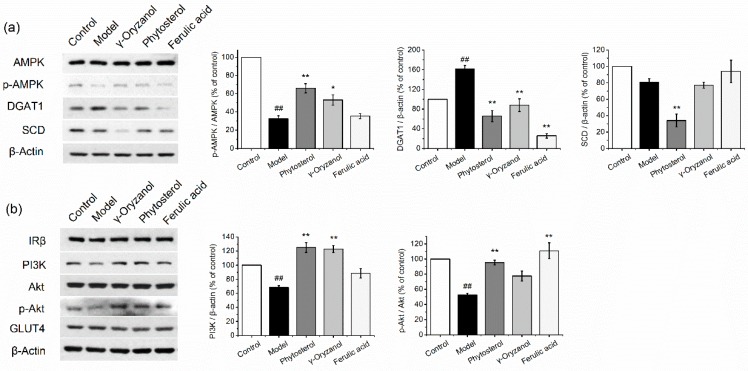
Western blot analysis of lipid (**a**) and glucose (**b**) metabolism-related protein expressions in HepG2 cells. Proteins were extracted from induction medium (10 mM glucose, 15 mM fructose and 1 mM FFA)-treated HepG2 cells with or without functional components (100 μM). 1% BSA in incomplete DMEM was selected as the control. Data are presented as mean ± SEM (*n* = 3). ## *p* < 0.01 vs. control. * *p* < 0.05 vs. model. ** *p* < 0.01 vs. model.

Expressions of lipid/glucose metabolism-related proteins were examined using SDS-PAGE in an effort to clarify the protein targets of γ-oryzanol, phytosterol and ferulic acid in regulating the lipid/glucose metabolism. Expression of lipid metabolism-related proteins were demonstrated in Figure 2a. AMPK is the center for energy homeostasis [23]. Total AMPK expression did not differ among all the groups. Fructose combined with FFA treatment-induced reduction in AMPK phosphorylation in the model cells, which was reversed by phytosterol and γ-oryzanol treatments with an elevation of 102.4% and 63.2% in the p-AMPK/AMPK ratio, as compared to the model cells (*p* < 0.05). DGAT1 is the key enzyme in the biosynthesis of TG. In model cells, the expression of DGAT1 significantly increased, which indicated an enhancement of TG synthesis. Ferulic acid treatment significantly downregulated the DGAT1 expression by as much as 84.0% compared to the model cells (*p* < 0.05), which were even lower than the control cells. Phytosterol and γ-oryzanol could also markedly decrease the expression of DGAT1 (*p* < 0.05). SCD is an enzyme involved in the synthesis of fatty acids. Interestingly, although no difference was observed between the control and model cells, phytosterol markedly decreased SCD expression by 57.9%, as compared to the model cells (*p* < 0.05).

Expressions of proteins from the insulin signaling pathway were shown in Figure 2b. Fructose combined with the FFA treatment did not change the expression of IRβ, but decreased the expression of PI3K, which might impair insulin signaling. Phytosterol and γ-oryzanol significantly reversed the decrease in the PI3K expression and induced an approximately 82% increase in PI3K expression as compared to model cells (*p* < 0.05), which were even higher than the normal cells. Akt is a downstream enzyme of PI3K that promotes GLUT4 translocation. Significantly decreased phosphorylation of Akt was detected in model cells compared to normal cells (*p* < 0.05), indicating an impairment in insulin sensitivity. All of the three compounds increased their insulin sensitivity by elevating p-Akt/Akt, among which phytosterol and ferulic acid exerted significant effects by increasing 81.2% and 110.8% of p-Akt/Akt ratio, respectively, as compared to model cells (*p* < 0.05). The expression of GLUT4 did not change among all the groups. Collectively, this in vitro study indicates a potential beneficial role of γ-oryzanol, phytosterol and ferulic acid in regulating glucose/lipid metabolic disorders.

### 3.3. Effect of Enriched Flour on Body Weight, Food Intake and Organ Indices of Rats

Diets rich in fats and fructose have been shown to contribute to several pathologies and have been frequently used in studies seeking to induce metabolic dysfunctions in vivo [24]. Therefore, we next studied the effects of γ-oryzanol, phytosterol and ferulic acid-enriched wheat flour on obesity-associated lipid/glucose metabolic disorder in rats fed by HFFD for 16 weeks. The results revealed that HFFD-feeding induced lipid and glucose metabolic disorder, such as abdominal obesity, dyslipidemia, hyperglycemia, insulin resistance and hepatic steatosis in rats (model group) (Table 2), indicating a successful establishment of the rat model. At the end of the study, body weight and tissue index were determined in the tested groups. As shown in Table 2, the rats in the model group showed a 32.9% increase in body weight gain compared to our control group (*p* < 0.05). OZ-90F and FA-90F reduced the body weight gain by 13.2% and 15.3%, respectively, compared with the model group (*p* < 0.05). Moreover, the body weight of FA-90F group was not significantly different from control rats (*p* > 0.05). There was no change in energy intake among all of the HFFD-fed groups. The HFFD-feeding resulted in a 16.4% increase in the liver index, and about 2-fold of fat indices in model rats compared with control rats, indicating that the model rats developed abdominal obesity. FA-90F reduced the liver index to the normal level seen in the control group. Moreover, FA-90F caused 32.1%, 27.1%, and 25.0% reduction in the perirenal, epididymal, and retroperitoneal fat indexes when compared with those in model rats (*p* < 0.05). 

### 3.4. Effect of Enriched Flour on Blood Lipid Profiles of Rats

**Figure 3:**
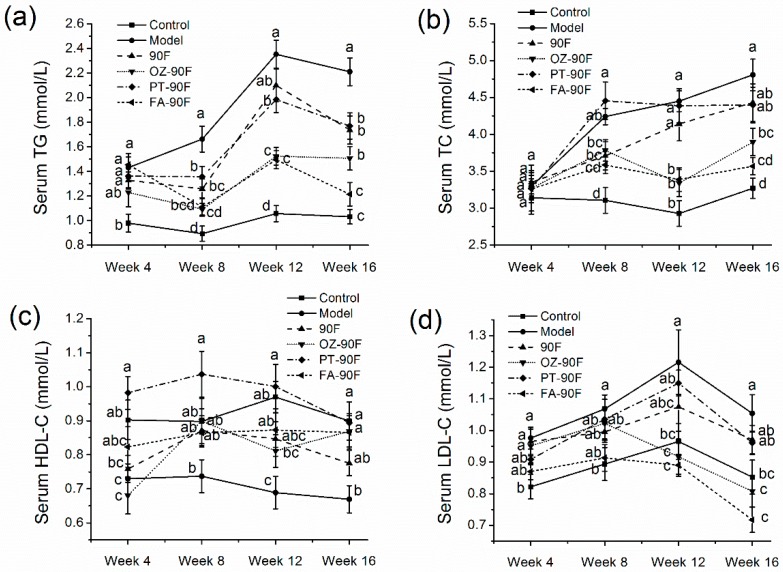
Effect of enriched flour on serum lipid profiles in rats. Blood samples were collected from orbital venous at week 4, 8, 12 and 16 from 12 h fasted rats. (**a**) Serum TG level; (**b**) serum TC level; (**c**) serum HDL-C level; (**d**) serum LDL-C level. Data are presented as mean ± SEM (*n* = 10). Mean values from the same week with different letters are significantly different (*p* < 0.05). 90F, 90% wheat flour; OZ-90F, γ-oryzanol enriched 90% wheat flour; PT-90F, phytosterol enriched 90% wheat flour; FA-90F, ferulic acid enriched 90% wheat flour.

After feeding on HFFD for 16 weeks, model rats showed severe dyslipidemia with considerably elevated levels of serum TG, TC, LDL-C, and a decreased level of HDL-C (Figure 3, *p* < 0.05). The model rats exhibited an elevated level of serum TG since week 4 (*p* < 0.05). A significant reversal in the elevated TG level was observed in week 8 by the consumption of the enriched flour, and was sustained until the end of the experiment (*p* < 0.05). OZ-90F and FA-90F showed significantly better results than the PT-90F and 90F by decreasing approximately 36.2% of the TG level compared with model rats at the week of 12 (*p* < 0.05). At the end of the study, the TG levels of model rats increased to approximately 2.3 times that of the control rats. FA-90F and OZ-90F exerted remarkable effects in lowering the levels of serum TG, with reductions of 45.2% and 32.1%, respectively, compared to the model group (*p* < 0.05). PT-90F and 90F exerted similar effects by decreasing 20.8% of the TG level (*p* < 0.05). HFFD induced a significant increase in the serum TC level since week 8 (*p* < 0.05), and the enriched flour beneficially regulated the elevated TC level at week 8 (*p* < 0.05). OZ-90F and FA-90F further significantly inhibited the increment in the TC level until the end of the experiment (*p* < 0.05), with reductions of 19.0% and 25.6%, respectively, compared with model rats at the end of the study. HFFD resulted in a significant reduction in the level of HDL-C since week 4 (*p* < 0.05). PT-90F markedly increased HDL-C level to the level numerically higher than the control rats until the week of 12. At the week of 8, all of the treatment groups could increase their HDL-C level to the normal level seen in the control group, and the effects sustained till the end of the study. At the end of the experiment, 90F, PT-90F and OZ-90F showed similar levels with control rats. Elevation in the levels of LDL-C is an indicator of a probable risk of myocardial infarction [25]. The LDL-C level was significantly elevated in the model group since week 4 (*p* < 0.05). FA-90F firstly showed potential in lowing the LDL-C level since week 8, however, the effect was not statistically significant (*p* > 0.05). At the week of 12, OZ-90F and FA-90F exerted remarkable effects in decreasing the LDL-C level to the level lower than the control rats, and the effects sustained until the end of week 16 (*p* < 0.05). LDL-C levels of OZ-90F and FA-90F were decreased by 22.9% and 31.4%, respectively, at the end of the experiment, as compared to the model rats (*p* < 0.05).

### 3.5. Effect of Enriched Flour on Glucose Metabolism and Insulin Resistance in Rats

**Figure 4:**
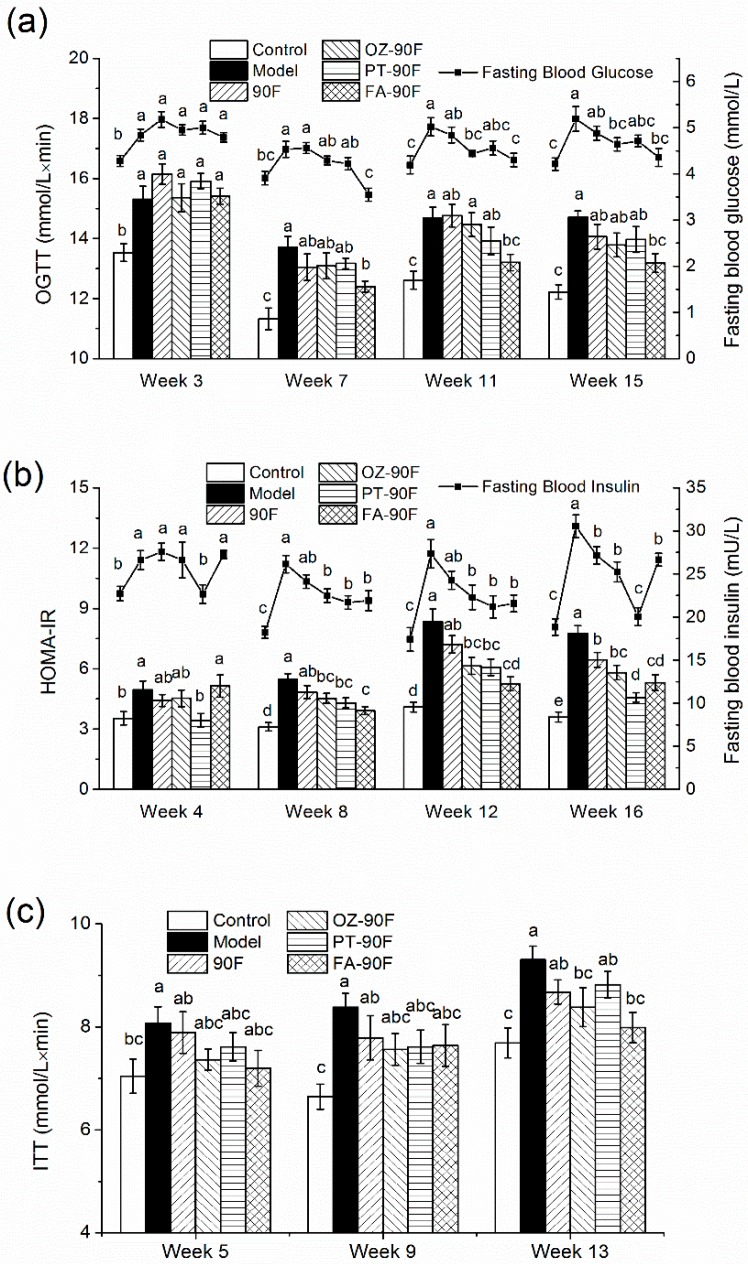
Effect of enriched flour upon glucose tolerance, insulin resistance, and insulin tolerance in rats. Fasting blood glucose and the AUC of the oral glucose tolerance test (**a**) were measured at week 3, 7, 11 and 15; Fasting blood insulin and HOMA-IR index (**b**) were measured at week 4, 8, 12 and 16. AUC of insulin tolerance test (**c**) was measured at week 5, 9 and 13. Data are presented as mean ± SEM (*n* = 10). Mean values with different letters from the same week are significantly different (*p* < 0.05). 90F, 90% wheat flour; OZ-90F, γ-oryzanol enriched 90% wheat flour; PT-90F, phytosterol enriched 90% wheat flour; FA-90F, ferulic acid enriched 90% wheat flour.

To examine the effect of enriched flours on glucose tolerance in HFFD-fed rats, OGTT was conducted at weeks 3, 7, 11, and 15. As indicated in Figure 4a, model rats developed increased FBG and AUC of OGTT since week 4, compared with those in control rats (*p* < 0.05), indicating an establishment of impaired glucose tolerance in model rats. FA-90F firstly and significantly improved FBG and OGTT since week 7 (*p* < 0.05), and showed a 16.0% decrease in FBG and a 10.4% decrease in AUC of OGTT compared with those in the model group at week 15. The OZ-90F group also significantly reversed its elevation in the FBG level since week 11 (*p* < 0.05). Other groups showed potency in regulating FBG and glucose tolerance, however the effects were not statistically significant (*p* > 0.05).

Insulin resistance in these rats was determined by measuring serum insulin and HOMA-IR. As shown in Figure 4b, the HFFD-feeding induced a significantly elevated serum insulin level and HOMA-IR since week 4, indicating that insulin resistance was developed in model rats. Insulin resistance in model rats worsened as the experiment continued, and finally resulted in 62.2% and 113.9% increase in fasting blood insulin and HOMA-IR at week 16, respectively, compared with the control rats. PT-90F firstly showed potency in lowering the blood insulin level and HOMA-IR among all of the treated groups (*p* < 0.05), and its effect sustained till the end of the experiment. OZ-90F and FA-90F exerted significant effects in decreasing the blood insulin level and HOMA-IR since week 8 (*p* < 0.05). At the end of the experiment, rats in the groups of 90F also exhibited significantly low levels of fasting blood insulin and HOMA-IR (*p* < 0.05). Among these groups, the PT-90F group reduced as much as 34.2% of fasting blood insulin compared with the model rats at week 16 (*p* < 0.05), which almost reaches the level of the control rats.

Moreover, PT-90F showed a marked improvement of insulin resistance with a decrease of 40.8% in the HOMA-IR index compared with the model rats (*p* < 0.05).

An insulin tolerance test (ITT) was conducted at weeks 5, 9, and 13 (Figure 4c). The enriched flour showed its potential to improve insulin tolerance at weeks 5 and 9 (*p* > 0.05). At the week of 13, OZ-90F and FA-90F significantly reduced the AUC of ITT with reductions of 9.9% and 14.1%, respectively, as compared to the model rats (*p* < 0.05).

### 3.6. Effect of Enriched Flour on Hepatic Steatosis, MDA Level and Hepatic Function of Rats

**Figure 5:**
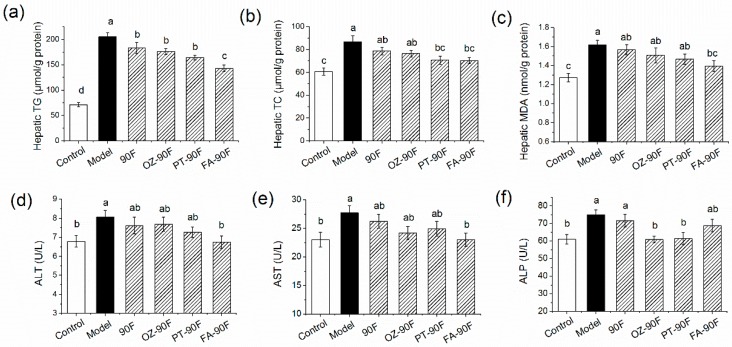
Effect of enriched flour on hepatic lipid and MDA accumulation and hepatic function in rats. At the end of the study, the hepatic tissue was dissected and homogenated for the measurement of hepatic TG (**a**), TC (**b**), and MDA (**c**). Blood samples were collected from orbital venous at week 16 from 12-h fasted rats for the measurement of serum ALT activity (**d**), AST activity (**e**), and ALP activity (**f**). Data are presented as mean ± SEM (*n* = 10). Mean values with different letters are significantly different (*p* < 0.05). 90F, 90% wheat flour; OZ-90F, γ-oryzanol enriched 90% wheat flour; PT-90F, phytosterol enriched 90% wheat flour; FA-90F, ferulic acid enriched 90% wheat flour.

To test the effects of enriched flour upon lipid accumulation in liver, the hepatic TG and TC levels were determined in these rats at the end of the study. As shown in Figure 5a,b, HFFD induced an approximately 3-fold increase in hepatic TG level and a 1.4-fold increase in hepatic TC level in model rats, as compared to the control rats (*p* < 0.05). FA-90F exhibited 40.7% and 19.0% reductions in hepatic TG and TC accumulation, respectively, compared with those in the model rats (*p* < 0.05). PT-90F and OZ-90F exerted similar effects by decreasing approximately 25% of hepatic accumulation (*p* < 0.05). These findings are consistent with changes in the liver index (Table 2).

As a marker of lipid peroxidation, the Hepatic MDA level significantly elevated in model rats after HFFD feeding (Figure 5c, *p* < 0.05). Although the enriched flours showed a potential to lower this MDA level, only the FA-90F group exhibited a significant effect with a reduction of 14.3% compared with the model rats (*p* < 0.05).

Hepatic fat accumulation is associated with liver dysfunctions [26], which is characterized by an elevated leakage of hepatic cellular enzymes [27]. Thus, hepatic function was determined by measuring the activities of ALT, AST and ALP in serum at the week of 16. 

HFFD combined with refined flour feeding caused a significant deterioration of hepatic function in model rats compared with control rats as indicated by the 18.9%, 20.5% and 23.0% increases in ALT, AST and ALP activities, respectively (Figure 5d–f, *p* < 0.05). However, the FA-90F treatment showed the most significant effect on normalizing ALT and AST activities to the levels in the control group (*p* < 0.05). On the other hand, OZ-90F was the most effective in reducing the ALP activity, which was an 18.9% reduction compared with model rats (*p* < 0.05).

### 3.7. Effect of Enriched Flour on Oxidative Stress and Hyperuricemia in Rats

**Figure 6:**
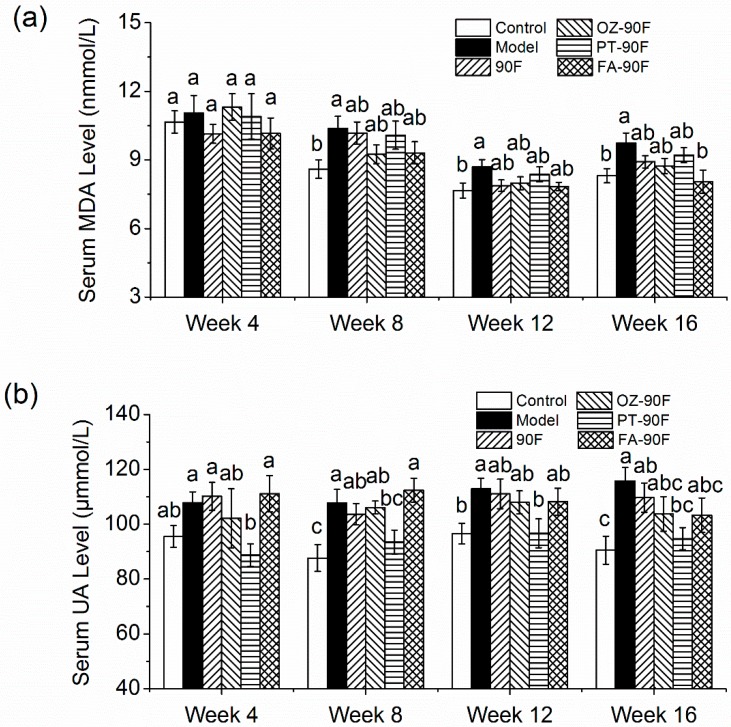
Effect of enriched flour on oxidative stress and hyperuricemia in rats. The same serum was used as Figure 3 for measurement of MDA (**a**) and UA (**b**) levels. Mean values with different letters from the same week are significantly different (*p* < 0.05). 90F, 90% wheat flour; OZ-90F, γ-oryzanol enriched 90% wheat flour; PT-90F, phytosterol enriched 90% wheat flour; FA-90F, ferulic acid enriched 90% wheat flour.

Next, we examined the effects of enriched flour on oxidative stress as judged by the measurement of serum MDA. The serum MDA level in model rats increased by 17.2% compared with control rats at the end of the study (*p* < 0.05). All of the treatment groups exhibited the tendency to decrease their MDA level since week 12 (*p* > 0.05), while at the week of 16, FA-90F significantly decreased the MDA level by 17.3% as compared with model rats (*p* < 0.05), which was even numerically lower than the control rats (Figure 6a).

Emerging data suggest that the elevated UA caused by increased fructose intake is actually one of the most important risk factors for the pathogenesis of metabolic syndrome and cardiovascular disease [28]. The present study revealed that model rats exhibited a 23.0% increase in serum UA level at the week of 8, which further increased by 27.9% at the end of the study compared to control rats (*p* < 0.05), indicating hyperuricemia was developed in model rats after HFFD feeding (Figure 6b). Interestingly, PT-90F remarkably suppressed the serum UA level throughout the experiment, which exhibited an 18.2% decrease in rats at week 16 compared with the model rats (*p* < 0.05). Other treatment groups also showed a potential to decrease this UA level, however, the effect was not statistically significant (*p* > 0.05).

### 3.8. Expression of Lipid/Glucose Metabolism-Related Proteins in Liver Tissue

**Figure 7:**
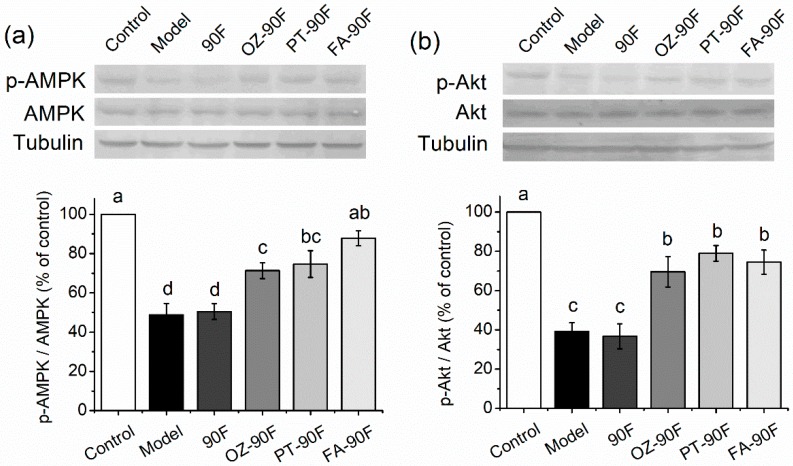
Western blot analysis of AMPK (**a**) and Akt (**b**) expressions in liver tissues. Proteins were extracted from the liver tissues of treated rats. Data are presented as mean ± SEM (*n* = 3). Mean values from the same week with different letters are significantly different (*p* < 0.05). 90F, 90% wheat flour; OZ-90F, γ-oryzanol-enriched 90% wheat flour; PT-90F, phytosterol-enriched 90% wheat flour; FA-90F, ferulic acid-enriched 90% wheat flour.

Expressions of AMPK, p-AMPK, Akt and p-Akt in the liver tissue were detected using SDS-PAGE. Results were shown in Figure 7. HFFD-induced reduction in AMPK phosphorylation in model rats, which was significantly reversed by OZ-90F, PT-90F and FA-90F with an elevation of 46.1%, 52.9% and 79.9%, respectively, in the p-AMPK/AMPK ratio, as compared to the model cells (*p* < 0.05). This result is consistent with the liver steatosis results (Figure 5). To examine the effect of enriched flour upon insulin resistance, a phosphorylation of Akt was detected. OZ-90F, PT-90F and FA-90F increased insulin sensitivity by elevating p-Akt/Akt ratio by 77.5%, 101.5%, and 89.9%, respectively, as compared to model cells (*p* < 0.05).

### 3.9. Histological Examination

**Figure 8:**
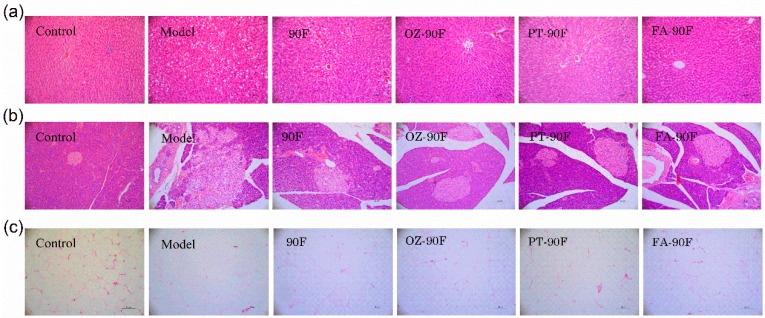
Histological examination. At the end of the study, the rats were dissected and the liver (**a**), pancreas (**b**) and epididymal fat (**c**) were collected for histological examination. Representative samples were stained with hematoxylin and eosin. The bar represents 94 μm. 90F, 90% wheat flour; OZ-90F, γ-oryzanol enriched 90% wheat flour; PT-90F, phytosterol enriched 90% wheat flour; FA-90F, ferulic acid enriched 90% wheat flour.

The liver, pancreas, and epididymal adipose tissue collected from these rats were subjected to histology to determine the role of the enriched flours in the HFFD-induced pathological alteration. As shown in Figure 8a, an examination of HE-stained liver sections revealed the typical architecture of the hepatic lobules in control rats. In contrast, the model rats showed degenerative changes in the hepatic lobules, characterized by lots of fat vacuoles depositing in hepatocytes. This was partially reduced by the OZ-90F and FA-90F treatments showing that less fat accumulated in the liver. The HE staining of the pancreas was shown in Figure 8b. The pancreatic islet in the model rats showed severe damages in its shape with inflammatory cell infiltration. The enriched flours greatly normalized the pancreatic structure. In line with the fat indices shown in Table 2, the HE staining of the adipose tissue showed a drastically larger and heterogeneous adipocyte size in model rats relative to control rats (Figure 8c). The FA-90F treatment showed a beneficial effect on suppressing the increase of the adipocyte size, which displayed almost uniform adipocyte size, and was comparable with our control group.

## 4. Discussion

Main phytochemicals (ferulic acid, γ-oryzanol, phytosterol) in the by-products of the refining process of grains are selected in the present study. Their effects on hepatic steatosis, glucose utilization and oxidative stress were evaluated on a cell model with lipid and glucose metabolic disorder. Our data demonstrated that all of the compounds showed the potential to alleviate hepatic steatosis. Phytosterol and γ-oryzanol significantly increased AMPK phosphorylation, which may lead to a concomitant inhibition of energy-consuming biosynthetic pathways and an activation of catabolic pathways [29]. The activation of AMPK is reported to be associated with a downregulation of the expression of the downstream transcription factor sterol regulatory element-binding protein 1c (SREBP1c), and the enzyme acetyl-CoA carboxylase (ACC), thereby decreasing hepatic cholesterol and fatty acid biosynthesis [30]. DGAT1 is an enzyme that catalyzes the final reaction in the known pathways of mammalian TG synthesis [31]. Reduced expression of DGAT1 is reported to be an effective way to prevent hepatic steatosis [32]. All of the compounds could downregulate the expression of DGAT1, among which, ferulic acid exerted a marked effect by decreasing the expression by approximately 85%, which might contribute to its lipid-lowering activity. SCD is an enzyme involved in the synthesis of fatty acids. Research studies have demonstrated that the knockdown of SCD prevents liver steatosis in wild-type mice [33]. Interestingly, only phytosterol decreased SCD expression, which might be a unique way for this phytosterol to regulate lipid metabolism. In the glucose absorption assay, phytosterol exhibited a marked improvement in insulin-induced glucose absorption, which might be associated with improved insulin resistance. The expressions of proteins from the insulin signaling pathway were detected using western blot. Consistent with the glucose absorption result, phytosterol significantly increased the PI3K expression and phosphorylation of Akt (*p* < 0.01), which is beneficial in glucose transportation. Therefore, phytosterol is capable of increasing the insulin sensitivity through activating the insulin signaling pathway. Significantly improved insulin resistance was observed in phytosterol-treated rats in the following in vivo study (Figure 4), as well as in a clinical trial as reported by Li et al. [34] γ-Oryzanol and ferulic acid also significantly elevated expressions of PI3K and phosphorylation of Akt, respectively (*p* < 0.01). Collectively, these results indicate a potential beneficial role of γ-oryzanol, phytosterol and ferulic acid in lipid/glucose metabolic disorder through different protein targets in vitro.

Based on the in vitro study, ferulic acid, phytosterol and γ-oryzanol were used to enrich wheat flour, in an effort to provide new thoughts in designing a functional staple food with the potential to regulate the lipid/glucose metabolism for obese people. We found that by replacing refined wheat flour in an HFFD with the enriched 90 flour, the risk factors for metabolic syndrome in rats are significantly diminished. The treated rats showed an improvement of hyperglycemia and glucose tolerance, which may be due to the increased utilization of glucose in the liver via an improvement of insulin sensitivity or a restoration of hepatic glucokinase and glycogen storage activity. Indeed, phosphorylation of Akt in liver tissue was significantly elevated by OZ-90F, PT-90F and FA-90F treatments, indicating improved insulin sensitivity (Figure 7). The improved glucose metabolism is also evidenced by western blot in the in vitro study (Figure 2). However, no significant improvement in glucose absorption was observed in ferulic acid and γ-oryzanol-treated cells (Figure 1). This is probably because the micro circumstance of the in vitro cell culture system is not as optimized as the in vivo study, and the treatment time is relatively short in the in vitro study.

FA-90F exerted the most comprehensive role in alleviating metabolic diseases. Ferulic acid is a well-studied antioxidant. It not only scavenges free radicals, but also increases the activity of enzymes that are responsible for scavenging free radicals, and inhibits enzymes that catalyze the production of free radicals. In the present study, the MDA level is measured as an indicator of oxidative stress. Indeed, ferulic acid exerted a significant effect in lowering MDA accumulation in HepG2 cells (Figure 1). FA-90F also significantly reduced hepatic MDA accumulation and serum MDA level in HFFD-fed rats, while the other groups reduced it numerically, but not statistically (Figure 5 and Figure 6). The alleviated oxidative stress might contribute to the hypoglycemia and insulin-sensitizing effects of FA-90F [35].

These enriched flours are also beneficial for regulating HFFD-induced liver steatosis, among which FA-90F exerted a significantly better effect in inhibiting the hepatic TG level than the other two groups (*p* < 0.05). The effect of FA-90F might be attributed to the decrease in DGAT1 expression as indicated in the in vitro study (Figure 2a), and the increased AMPK phosphorylation in liver tissue from FA-90F-treated rats (Figure 7a). Ferulic acid is also reported to decrease the expression of hepatic lipogenic genes, such as SREBP1c, fatty acid synthase (FAS) and ACC, thereby limiting the availability of the fatty acids required for a synthesis of hepatic triglycerides and also reduce the serum lipid levels in high-fat-diet fed obese mice [9]. Although phytosterol did not show any significant effect in lowering the hepatic lipid accumulation in HepG2 cells (Figure 1), the effect strengthened the in vivo study (Figure 5). The weak effect in the in vitro study might be due to the short treatment time. The hepatic lipid lowering effect of OZ-90F and PT-90F might be evidenced by reductions in expressions of p-AMPK, DGAT1, SCD by γ-oryzanol and phytosterol treatments in the in vitro study (Figure 2), and the downregulated p-AMPK in the liver tissue of treated rats (Figure 7).

Phytosterol is a well-studied compound with hypocholesterolemic effect. A supplementation of 2 g/day is recommended by the NCEP to reduce LDL-C and risk factors for cardiovascular diseases. However, in the present study, only a slight decrease in LDL-C was observed in the PT-90F-treated group. Racette et al. evaluated the effects of three phytosterol intakes on whole-body cholesterol metabolism in humans. Results showed that serum LDL-C declined significantly only with the highest phytosterol dose of 2 g/day [36]. Indeed, the effective dose of phytosterol for lowering the level of LDL-cholesterol has been reported to be about 1.5–3 g/day in humans [37]. In our study, the phytosterol intake was approximately 0.04 g/day per rat. According to the method of Reagan-Shaw et al., our dose is equivalent to 0.33-0.97 g/day for a 60 kg human [38], which is probably not high enough to appreciably influence cholesterol metabolism. Another study on high-fat-feeding mice revealed a significantly decreased serum cholesterol when the dose of phytosterol is 2% in mice chow [39], which is ten times that which was used in our study. Therefore the poor hypocholesterolaemic effect of phytosterol in the present study might be due to the low dose added in 90 flour. However, if a person consumes 300 g/day of PT-90F as a staple food, the intake of phytosterol would be 1.43 g/day. 

Meanwhile, there will be an extra of 167–437 mg/day phytosterol intake from common diets [40]. Thus the total intake of phytosterol should be enough to help lowering serum cholesterol according to previous researches [37].

Compared with PT-90F, FA-90F and OZ-90F both exerted remarkable effects in improving dyslipidemia, which might be due to significantly increased fecal lipid excretion (Son et al., 2010) [12]. Similar results were observed in previous studies in which ferulic acid and oryzanol supplementation resulted in improved dyslipidemia [12,27]. The doses of OZ and FA are equivalent to 0.50–1.46 and 0.16–0.48 g/day, respectively, when transferred from rats to a 60 kg human [38]. However, there are limited human data about the effect of OZ and FA, therefore research needs to be done to verify the effect of OZ and FA in a human study. It was suggested that ferulic acid inhibited the cholesterol synthesis by competitively inhibiting the activity of hydroxymethylglutaryl CoA reductase in the liver, and also by increasing the excretion of acidic sterol [41], thereby lowering the blood cholesterol levels. γ-Oryzanol is a mixture of ferulic acid esters of sterol and triterpene alcohols. The metabolite (ferulic acid) of oryzanol is more effective than oryzanol in regulating obesity and hyperlipidemia, which might partially contribute to the lipid-lowering effect of OZ-90F. These results indicate that FA-90F and OZ-90F might be more suitable to treat dyslipidemia than PT-90F at the same dose. Although PT-90F was not promising in improving hypercholesteremia in this study, it exerted a remarkable effect in regulating hyperinsulinemia and hyperuricemia. As far as we know, this is the first time that the improving effect of phytosterol on hyperinsulinemia has been reported. Given that UA will inhibit endothelial nitric oxide bioavailability, and nitric oxide is necessary in insulin-mediated glucose uptake [42], we hypothesize that the reduced UA level by phytosterol is a mechanism for the improved insulin sensitivity in rats fed with PT-90F. This hypothesis remains elusive. The underlying mechanism of phytosterol in regulating UA level needs to be further investigated.

## 5. Conclusions

**Figure 9:**
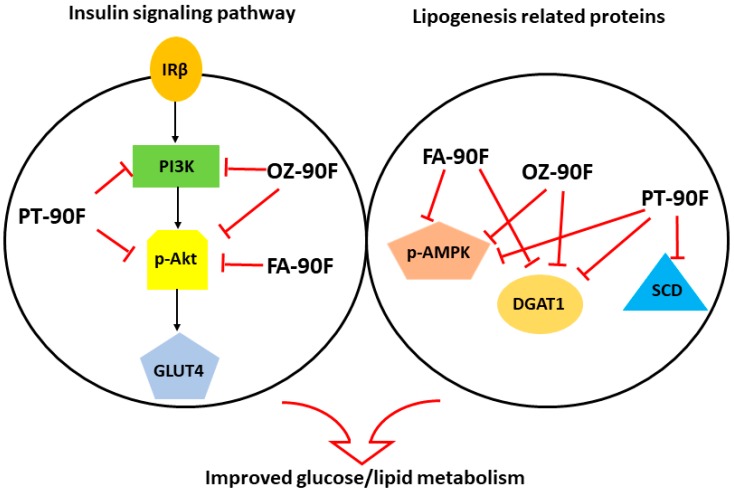
Potential pathway for enriched flours in regulating glucose/lipid metabolism.

Phytosterol, γ-oryzanol and ferulic acid were shown to target different proteins in regulating the lipid/glucose metabolism in an in vitro cellular model. These compounds alleviated lipid accumulation and insulin resistance in HepG2 cells through the elevation in PI3K expression and the phosphorylations of Akt and AMPK, and reduction in the expressions of DGAT1 and SCD (Figure 9). By replacing refined wheat flour in a HFFD with the γ-oryzanol/phytosterol/ferulic acid-enriched flour, the lipid/glucose metabolic disorder in rats are significantly improved, as evidenced by an improved lipid profile, hepatic steatosis, glucose/insulin tolerance, insulin resistance, oxidative stress, hepatic function and hyperuricemia. The present study provided evidence that these enriched flours can serve as a health-promoting functional food to reduce the risk factors for metabolic diseases in rats, among them, ferulic acid-enriched flour showed a more comprehensive effect. The effect needs to be further explored in human studies.

## Figures and Tables

**Table 1:** Composition of diets.

	Normal Diet	HFFD
Corn powder (g/100 g)	25.0	
Wheat middling (g/100 g)	20.0	
Refined wheat flour (g/100 g)	21.9	42.0
Soybean meal (g/100 g)	20.0	8.0
Chicken powder (g/100 g)		8.0
Fructose (g/100 g)		18.0
Lard (g/100 g)		20.0
Sodium cholate (g/100 g)		0.2
Calcium bicarbonate (g/100 g)	1.0	1.0
Mountain flour (g/100 g)	1.6	0.8
Miscellaneous meal (g/100 g)	4.5	
Plant oil (g/100 g)	2.0	
Fish powder (g/100 g)	2.0	
Vitamin mix (g/100 g)	2.0	2.0
Energy (kJ/g)	13.9	18.9

HFFD: high-fat-fructose diet.

**Table 2:** Effect of enriched flour on body weight, food intake, energy intake, and organ indices in HFFD-fed rats.

	Initial BW (g)	Final BW (g)	Food Intake (g/day)	Energy Intake (kJ/day)	Liver index (g/100 g BW)	Kidney Index (g/100 g BW)	Spleen Index (g/100 g BW)	Fat index (g/100 g BW)		
								Perirenal Fat	Epididymal Fat	Retroperitoneal Fat
Control	227.3 ± 5.3 ^a^	531.9 ± 15.8 ^c^	24.54 ± 0.18 ^a^	341.28 ± 2.54 ^b^	2.07 ± 0.04 ^b^	0.50 ± 0.01 ^a^	0.113 ± 0.004 ^a^	0.58 ± 0.03 ^c^	1.58 ± 0.09 ^d^	1.63 ± 0.09 ^d^
Model	226.1 ± 5.7 ^a^	631.0 ± 11.3 ^a^	20.98 ± 0.44 ^b^	396.36 ± 8.28 ^a^	2.41 ± 0.08 ^a^	0.44 ± 0.02 ^b^	0.107 ± 0.005 ^a^	1.12 ± 0.07 ^a^	3.10 ± 0.10 ^a^	3.60 ± 0.18 ^a^
90F	228.4 ± 2.2 ^a^	607.9 ± 19.6 ^ab^	20.19 ± 0.55 ^b^	376.75 ± 10.20 ^a^	2.28 ± 0.10 ^ab^	0.47 ± 0.02 ^ab^	0.101 ± 0.005 ^a^	0.98 ± 0.06 ^a^	2.73 ± 0.19 ^ab^	3.36 ± 0.18 ^ab^
OZ-90F	228.6 ± 5.9 ^a^	580.0 ± 11.2 ^b^	20.74 ± 0.51 ^b^	386.12 ± 9.51 ^a^	2.22 ± 0.05 ^ab^	0.47 ± 0.02 ^ab^	0.110 ± 0.003 ^a^	0.97 ± 0.05 ^a^	2.59 ± 0.12 ^bc^	3.02 ± 0.19 ^bc^
PT-90F	227.9 ± 6.6 ^a^	606.5 ± 18.5 ^ab^	21.49 ± 0.25 ^b^	400.47 ± 4.70 ^a^	2.27 ± 0.06 ^ab^	0.45 ± 0.01 ^b^	0.111 ± 0.006 ^a^	1.07 ± 0.04 ^a^	2.95 ± 0.13 ^ab^	3.45 ± 0.11 ^ab^
FA-90F	229.3 ± 3.1 ^a^	572.4 ± 17.8 ^bc^	20.01 ± 0.12 ^b^	373.17 ± 2.30 ^a^	2.06 ± 0.08 ^b^	0.48 ± 0.01 ^ab^	0.100 ± 0.003 ^a^	0.76 ± 0.04 ^b^	2.26 ± 0.14 ^c^	2.70 ± 0.18 ^c^

Results represent mean values ± SEM (*n* = 10). Mean values with different superscript letters are significantly different (*p* < 0.05). BW: Body weight.
